# Supplementary material for: Rationality and cognitive bias in captive gorillas’ and orang-utans’ economic decision-making
Source: PLoS One. 2022 Dec 14;17(12):e0278150. doi: 10.1371/journal.pone.0278150 (PMC9749992; doi:10.1371/journal.pone.0278150)
Supplement: S1 File — (PDF) [file pone.0278150.s001.pdf]

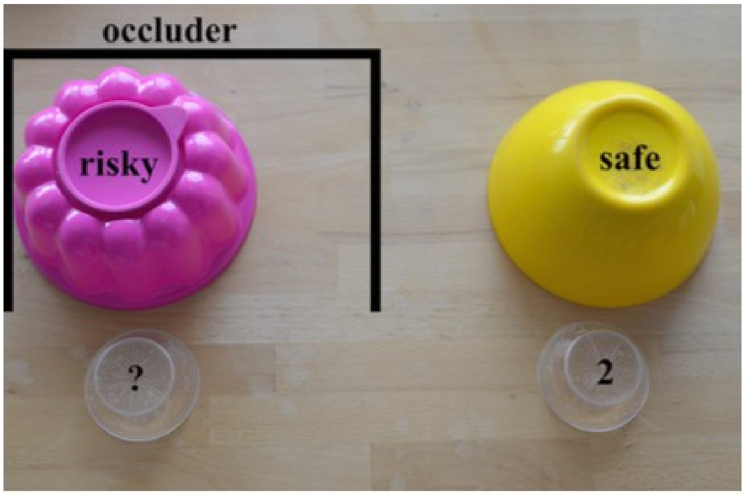

Fig S1. Apparatus for Experiment 1. Safe yellow cup (on the right), risky pink cup (on the left) under the occluder. In front of each cup there is the corresponding saucer where the experimenter puts, or pretends to put, the corresponding reward (in full view of the subject for the safe saucer, under the occluder for the risky one). Sides of safe and risky options are counterbalanced across sessions. The safe saucer contains 2 pieces of food. The risky saucer can contain 2, 4 or 6 pieces of food with different probabilities according to the session (P=1, 0.5, 0.33 or 0.25), see table 1.

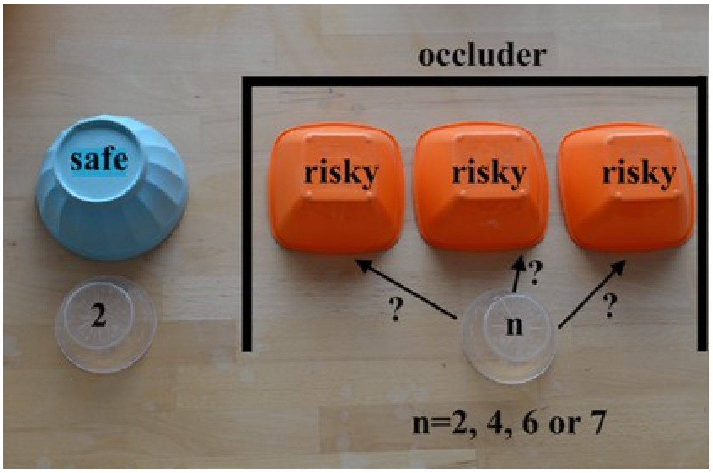

Fig S2. Apparatus for Experiment 2. Safe blue cup (on the left), risky set of orange cups (on the right) under the occluder. The experimenter puts the two saucers on top of the occluder, then fills them with the corresponding reward and hides them under the corresponding cups. Sides of safe and risky options are counterbalanced across sessions. The safe saucer contains 2 pieces of food. The risky saucer contains 2, 4, 6 or 7 pieces of food depending on the session. The number of risky cups varies between 1 to 4. See table 2 for all values of the risky option.

Run 1

Reward : **Number of cups :**

N=2	1	3	2	4	1	3	2	4	1	3
N=7	3	2	4	1	3	2	4	1	4	2
N=4	2	3	1	4	2	4	3	1	4	2
N=6	3	1	4	1	3	2	1	4	2	3
N=6	4	2	3	2	1	4	2	3	1	4
N=4	2	4	3	3	1	2	4	1	3	1
N=2	3	1	2	4	4	1	2	3	4	2
N=7	4	3	1	2	4	3	1	1	3	2

Run 2

N=7	3	1	2	4	4	1	2	3	4	2
N=6	2	4	3	3	1	2	4	1	3	1
N=4	4	2	3	2	1	4	2	3	1	4
N=6	2	3	1	4	2	4	3	1	4	2
N=4	3	1	4	1	3	2	1	4	2	3
N=2	4	3	1	2	4	3	1	1	3	2
N=7	1	3	2	4	1	3	2	4	1	3
N=2	3	2	4	1	3	2	4	1	4	2

Fig S3. Experimental procedure for Experiment 2, for one subject and one type of reward. Each line of the table shows one session (10 trials). The first column shows the quantity of reward that was tested for each session, and the other 10 columns show the number of risky cups for each trial of the session. For each run of the experiment, we ran 8 sessions (2 sessions per reward quantity). Each combination P (probability to win, i.e 1/number of cups) x V (reward quantity) was tested 5 times (grey cells for the combination P=0.25 x V=2).

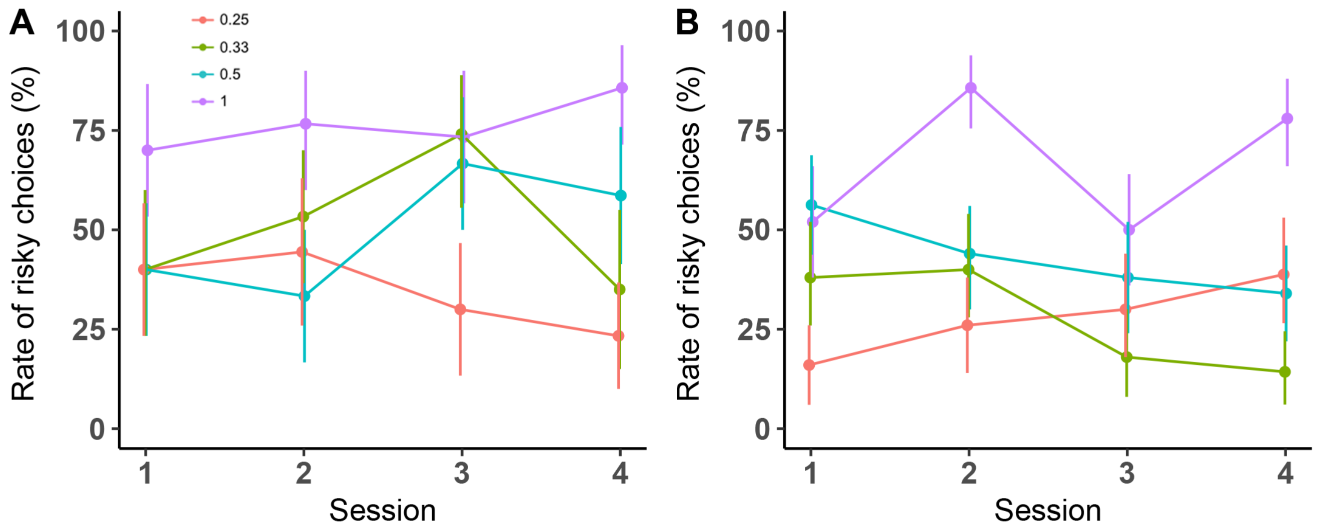

Fig S4. Impact of the session on subjects' perception of the probability of the risky option in Experiment 1. Mean level of risky choice for gorillas (A) and orang-utans (B) for each session, as a function of the probability of the risky option. Error bars indicate 95% confidence intervals.

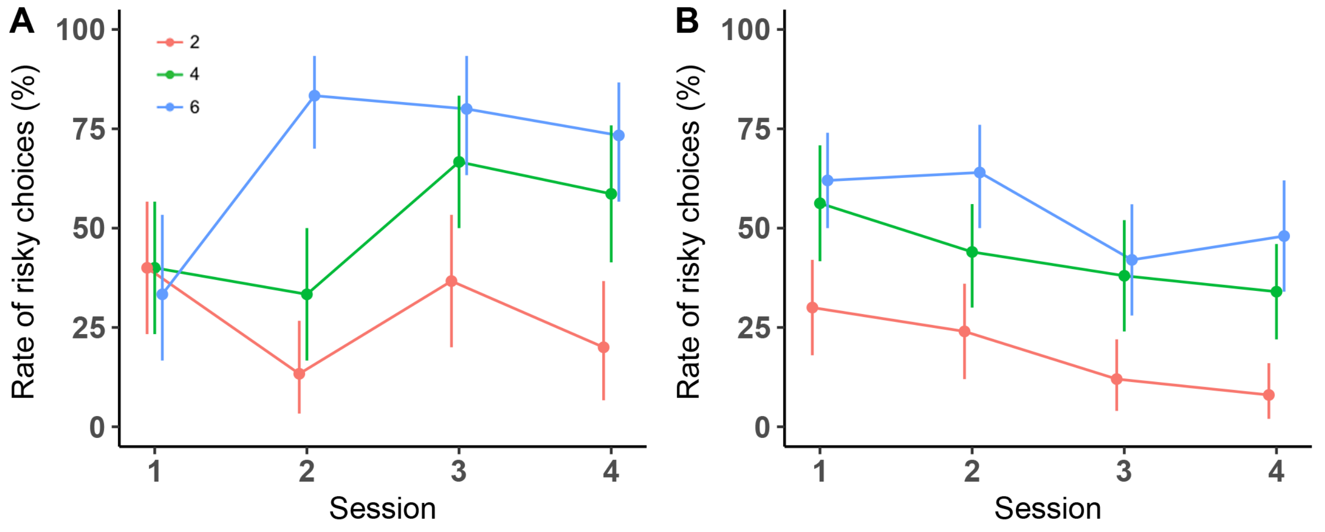

Fig S5. Impact of the session on subjects' perception of the value of the risky option in Experiment 1. Mean level of risky choice for gorillas (A) and orang-utans (B) for each session, as a function of the value of the risky option. Error bars indicate 95% confidence intervals.

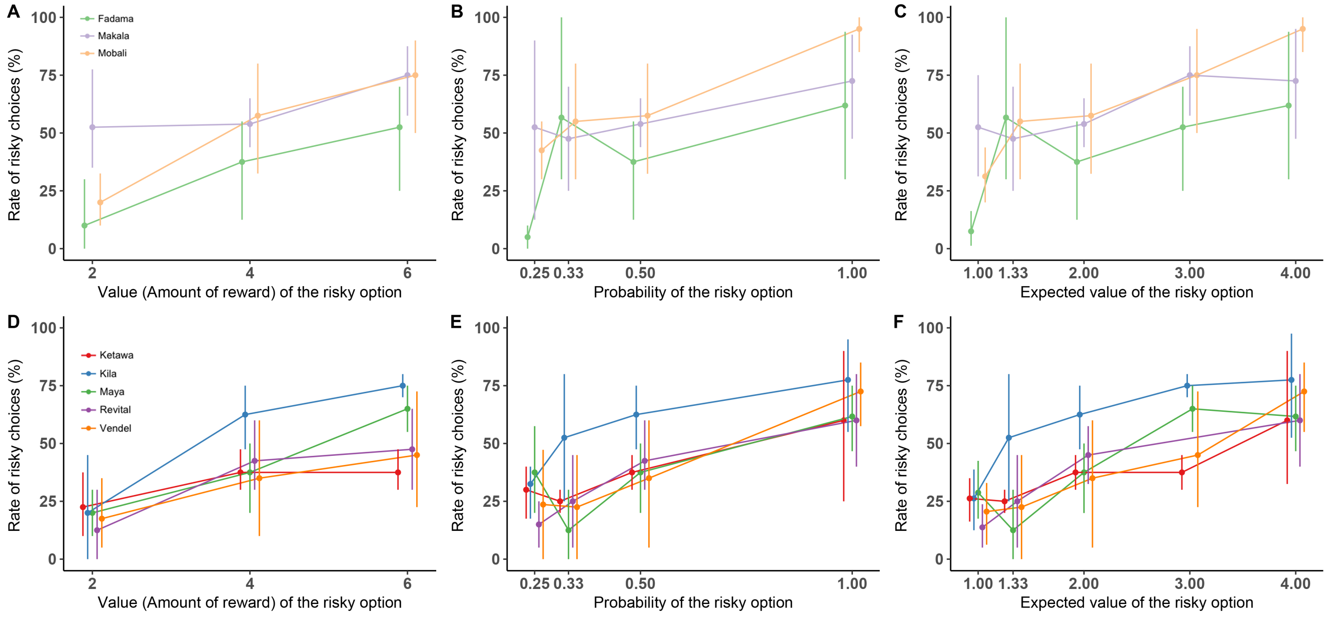

Fig S6. Individual performance in Experiment 1. Mean level of risky choice for each individual in Experiment 1, as a function of the value of the risky option (A for gorillas and D for orang-utans), the probability of the risky option (B for gorillas and E for orang-utans), or the expected value of the risky option (C for gorillas and F for orang-utans). Error bars indicate 95% confidence intervals.

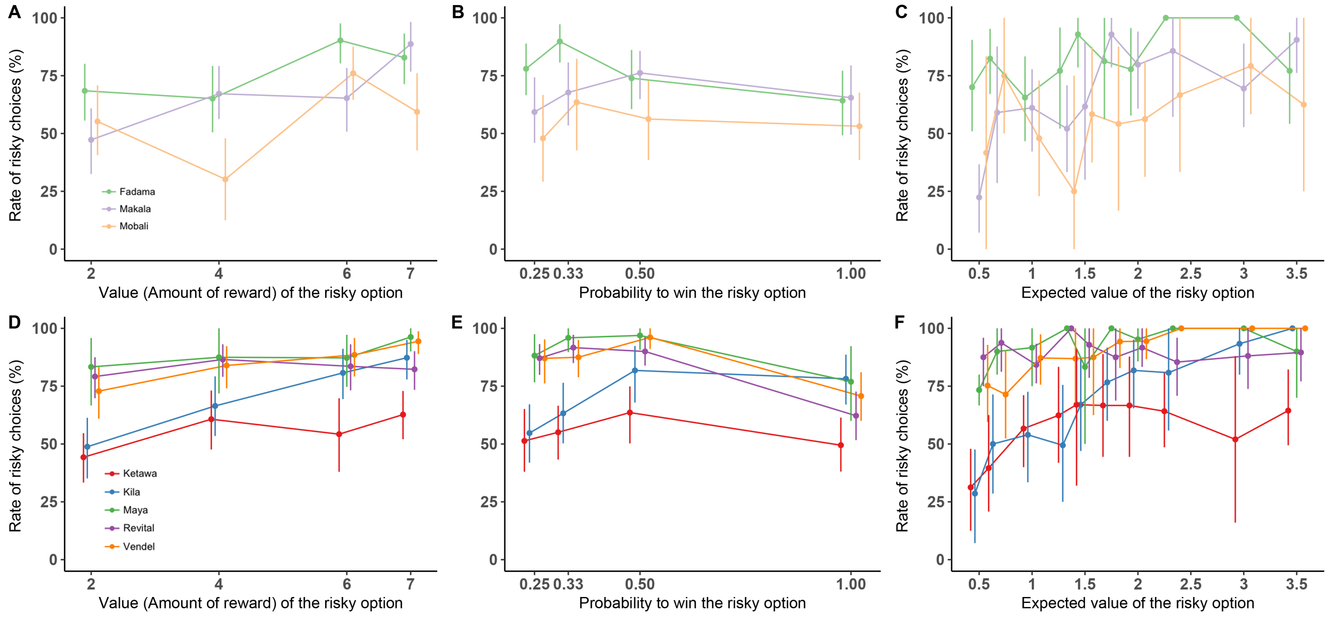

Fig S7. Individual performance in Experiment 2. Mean level of risky choice for each individual in Experiment 2, as a function of the value of the risky option (A for gorillas and D for orang-utans), the probability of the risky option (B for gorillas and E for orang-utans), or the expected value of the risky option (C for gorillas and F for orang-utans), for both types of reward combined. Error bars indicate 95% confidence intervals.

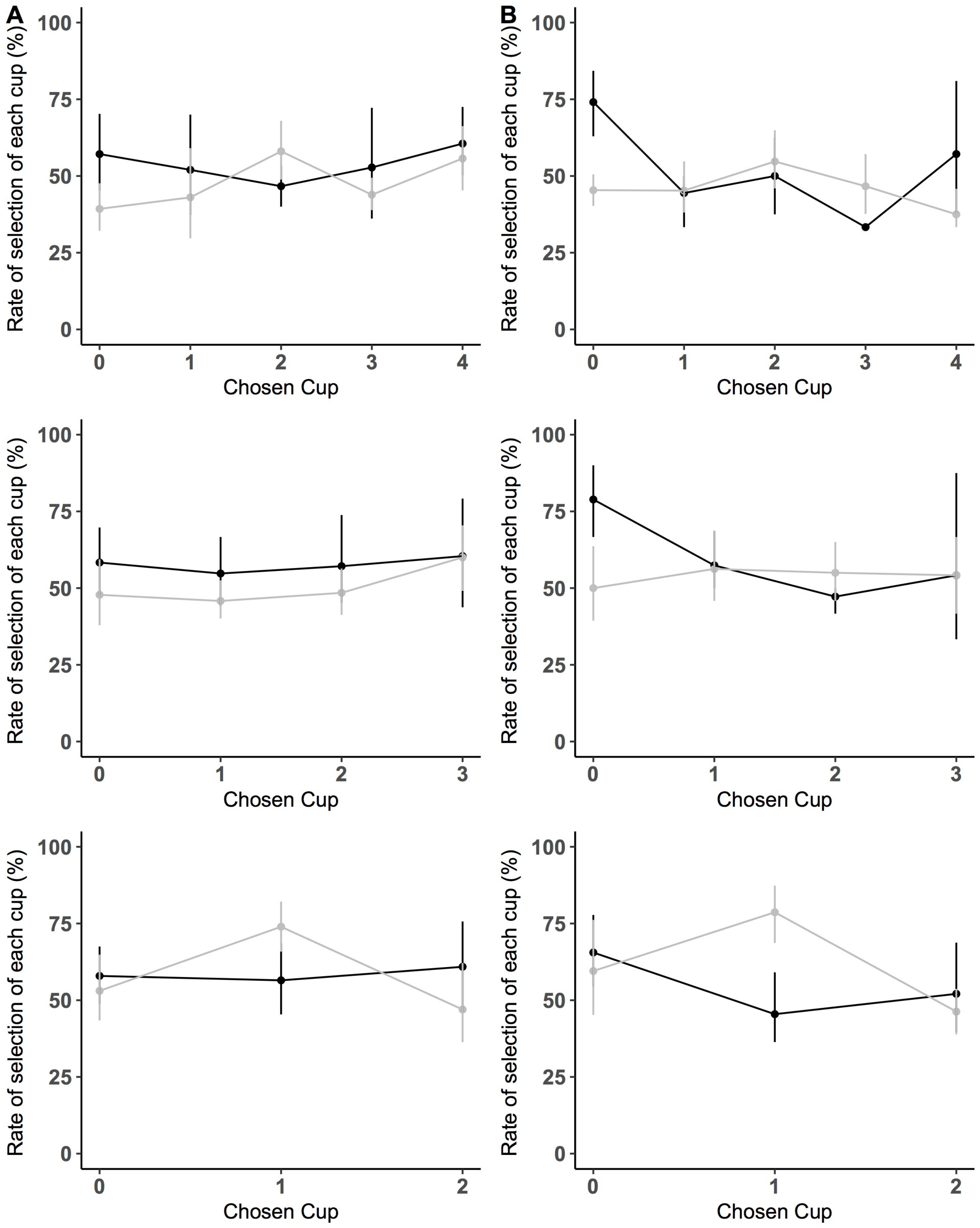
A
B
Rate of selection of each cup (%)
100
75
50
25
0
0
1
2
3
4
Chosen Cup

Fig S8. Positional preferences in Experiment 2 (for trials with more than 1 risky cups, for both types of reward). Mean percentage of selection of each cup, numbered from 0 (the cup on the far left of the subject) to 4 (the cup on the far right of the subject, when there are 4 risky cups), 3 (respectively, when there are 3 risky cups) or 2 (respectively, when there are 2 risky cups), for gorillas (black) and orang-utans (grey), when the safe cup is a the left side of the trolley (A) or at the right of the trolley (B). Error bars indicate 95% confidence intervals.

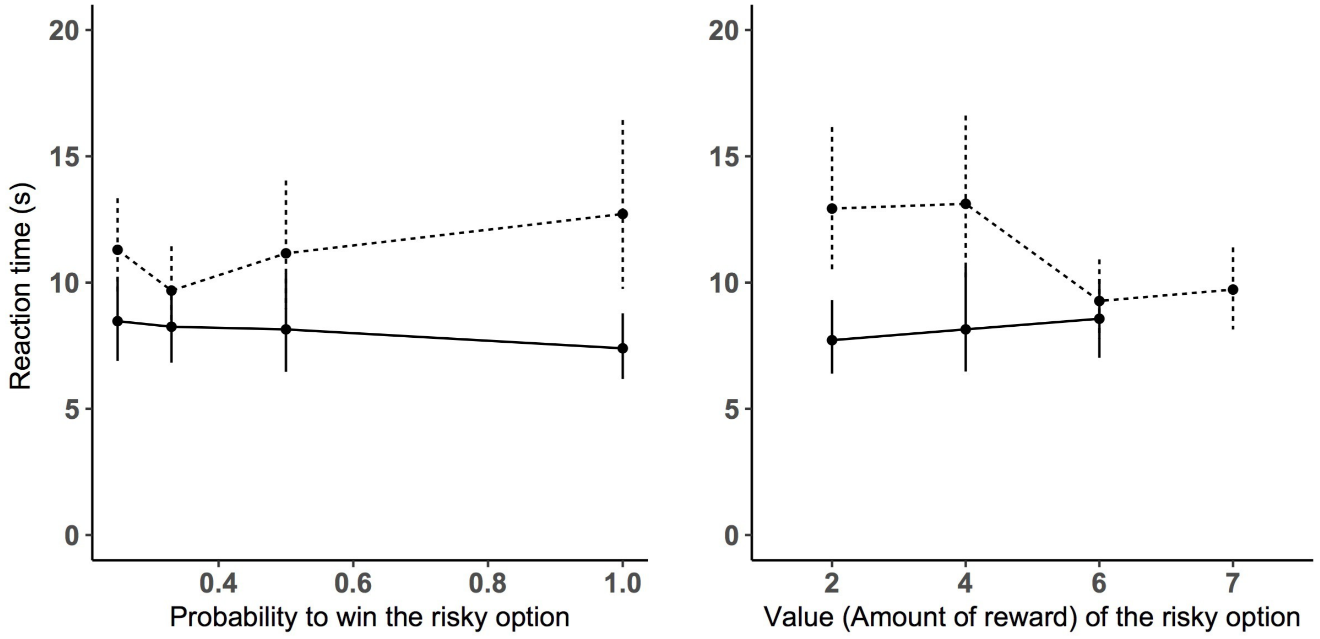

Fig S9. Response times in Experiment 1 (solid) and Experiment 2 (dotted, low-valued reward). Error bars indicate 95% confidence intervals.

Table S1 List of subjects for each experiment

Name	Species	Sex	Age	Experiments
Ketawa	Orangutan	Female	Juvenile (6 yo)	E2-/E1/E2+
Revital	Orangutan	Female	Adult (19 yo)	E1/E2-/E2+
Vendel	Orangutan	Male	Adult (19 yo)	E2-/E1/E2+
Kila	Orangutan	Female	Adult (18 yo)	E2-/E1/E2+

Maya	Orangutan	Female	Adult (13 yo)	E1/ E2-
Bagus	Orangutan	Male	Adult (17 yo)	E1/E2+
Fadama	Gorilla	Female	Adult (36 yo)	E2-/E1/E2+
Makala	Gorilla	Female	Juvenile (4 yo)	E1/E2-/E2+
Mobali	Gorilla	Male	Juvenile (4 yo)	E1/E2-
Adira	Gorilla	Female	Adult (13 yo)	E1

E1: Experiment 1; E2-: Experiment 2 with low-valued food: E2+: Experiment 2 with high-valued food. Subjects with their name in italic where removed from the study as they did not completed E2- after over 12 months from the beginning of the experiment.

Table S2. Random structure of the full, final and null model for Experiment 1. The standard deviations are indicated in the table for the full, the final and null model.

grouping	variable	full model	final model	null model
subject	intercept	0.242	0.262	0.649
	probability of the risky option	0.009	0.007	2.461
	value of the risky option	0.076	0.076	0.434
session within subject	intercept	0.203	0.148	0.23
	probability of the risky option	0.591	0.676	0.517
	value of the risky option	0.002	0.001	0.098

Table S3. Estimates of the full, final and null model investigating the impact of economic, specific and experimental parameters on risky choices in Experiment 1. The table contains parameter estimates +/- s.e. for the final model. For categorical predictors (species, session, and side of the safe cup), the tested levels are indicated in parentheses.

	full model	final model	null model
intercept	-0.22 +/- 0.71	-0.24 +/- 0.65	-3.12 +/-0.3302
species (Orang-utan)	-1.87 +/- 0.90	-1.85 +/- 0.80	
side of the safe cup (left of the	-0.83 +/- 0.14	-0.83 +/- 0.14	

subject)			
risky probability	1.67 +/- 0.81	1.72 +/- 0.49	
risky value	-0.08 +/- 0.14	-0.08 +/- 0.14	
session (2)	-4.19 +/- 1.16	-4.69 +/-1.08	
session (3)	-2.27 +/- 0.99	-2.12 +/- 0.92	
session (4)	-4.35 +/- 1.05	-4.04 +/- 0.96	
species (Orang) :session 2	2.94 +/- 1.41	3.67 +/- 1.25	
species (Orang) :session 3	1.27 +/- 1.27	1.04 +/- 1.12	
species (Orang) :session 4	2.14 +/- 1.22	1.65 +/- 1.16	
risky probability : session 2	0.39 +/- 1.21	1.52 +/- 0.75	
risky probability : session 3	0.39 +/- 1.13	0.04 +/- 0.69	
risky probability : session 4	2.83 +/- 1.26	2.07 +/- 0.73	
species (Orang) : risky probability	0.07 +/- 0.99		
risky option : session 2	1.06 +/- 0.24	1.07 +/- 0.24	
risky option : session 3	0.61 +/- 0.99	0.61 +/- 0.21	
risky option : session 4	0.71 +/- 0.21	0.71 +/- 0.21	
species (Orang) : risky option	0.43 +/- 0.18	0.44 +/- 0.19	
species (Orang) : risky probability : session 2	1.7 +/- 1.51		
species (Orang) : risky probability : session 3	-0.54 +/- 1.41		
species (Orang) : risky probability : session 4	-1.17 +/- 1.53		
species (Orang) : risky option : session 2	-0.95 +/- 0.29	-0.97 +/- 0.29	
species (Orang) : risky option : session 3	-0.56 +/- 0.26	-0.56 +/- 0.26	
species (Orang) : risky option : session 4	-0.54 +/- 0.26	-0.54 +/- 0.26	

Table S4. Estimated slopes of the final model for the economic parameters (probability of the risky option, value of the risky option) in Experiment 1.

Trends for the predictor: probability of the risky option

session	estimate	standard error	95% confidence interval lower	95% confidence interval upper	z.ratio	p-value
1	0.41	0.11	0.18	0.63	3.52	<.005
2	0.77	0.14	0.50	1.03	5.63	<.005
3	0.37	0.10	0.17	0.58	3.60	<.005
4	0.84	0.12	0.60	1.09	6.88	<.005

Trends for the predictor: value of the risky option

species	session	estimate	standard error	95% confidence interval lower	95% confidence interval upper	z.ratio	p-value
gorilla	1	-0.02	0.03	-0.09	0.05	-0.53	0.59
	2	0.23	0.05	0.14	0.32	4.94	<.001
	3	0.12	0.04	0.05	0.20	3.33	<.001
	4	0.15	0.04	0.07	0.23	3.87	<.001
orang-utan	1	0.08	0.03	0.03	0.14	3.12	<.005
	2	0.11	0.03	0.05	0.16	3.85	<.001
	3	0.08	0.02	0.03	0.12	3.39	<.001
	4	0.11	0.03	0.06	0.16	4.29	<.001

Table S5. Random structure of the full, final and null model for Experiment 2. The standard deviations are indicated in the table for the full, the final and null model. Only trials with P<1 were considered.

grouping	variable	full model	final model	null model
subject	intercept	0.80	0.81	1.22
	probability of the risky option	0.33	0.53	2.59
	value of the risky option	0.04	0.04	0.30
session within subject	intercept	0.01	0.01	0.33
	probability of the risky option	0.46	0.60	0.35
	value of the risky option	0.07	0.08	0.11

Table S6. Estimates of the full, final and null model investigating the impact of economic, specific and experimental parameters on risky choices in Experiment 2. The table contains parameter estimates +/- s.e. for the final model. For categorical predictors (species, session, and side of the safe cup), the tested levels are indicated in parentheses. Only trials with P<1 were considered.

	full model	final model	null model
intercept	-0.93 +/- 0.94	-1.21 +/-0.62	-0.37 +/- 0.53
species (Orang-utan)	0.75 +/- 1.2	0.76 +/- 0.71	
side of the safe cup (left of the subject)	-0.51 +/- 0.14	-0.48 +/- 0.14	
risky probability	-0.70 +/- 1.84	2.52 +/- 0.69	
risky value	0.48 +/- 0.11	0.29 +/- 0.04	
reward (vegetable)	-0.40 +/- 0.14	-0.49 +/- 0.14	
session (2)	0.51 +/- 1.15	0.21 +/- 0.27	
session (3)	-0.14 +/- 1.14	0.51 +/- 0.27	
session (4)	1.72 +/- 1.30	0.84 +/- 0.28	
species (Orang) :session 2	-0.94 +/- 1.52		
species (Orang) :session 3	-0.04 +/- 1.50		
species (Orang) :session 4	-1.64 +/- 1.65		
risky probability : session 2	2.37 +/- 2.62		
risky probability : session 3	4.15 +/- 2.65		
risky probability : session 4	2.05 +/- 2.97		
species (Orang) : risky probability	4.37 +/- 2.47		
risky option : session 2	-0.26 +/- 0.15		
risky option : session 3	-0.20 +/- 0.15		
risky option : session 4	-0.23 +/- 0.18		
species (Orang) : risky option	-0.34 +/- 0.15		
species (Orang) : risky probability : session 2	-2.63 +/- 3.53		
species (Orang) : risky probability : session 3	-3.71 +/- 3.6		

species (Orang) : risky probability : session 4	-2.31 +/- 3.89		
species (Orang) : risky option : session 2	0.44 +/- 0.20		
species (Orang) : risky option : session 3	0.36 +/- 0.21		
species (Orang) : risky option : session 4	0.47 +/- 0.22		

Table S7. Estimated marginal means and standard errors by session, and estimated marginal means by session at the indifference point (EV=2) for both reward types (low-valued: vegetable, and high-valued: grape), with the corresponding lower and upper limits of the 95% confidence interval, calculated with the final model for Experiment 2.

				**95% confidence interval**			**95% confidence interval**	
session	EMMs (all trials)	SE (all trials)	EMMs (EV=2, Reward: Low)	lower	upper	EMMs (EV=2, Reward: High)	lower	upper
1	0.72	0.07	0.68	0.52	0.85	0.76	0.62	0.9
2	0.76	0.07	0.73	0.58	0.88	0.8	0.67	0.92
3	0.8	0.06	0.78	0.65	0.91	0.84	0.73	0.95
4	0.85	0.05	0.83	0.72	0.95	0.88	0.79	0.97

Table S8. Random structure of the full, final and null model for Experiment 1 and 2 combined. The standard deviations are indicated in the table for the full, the final and null model. Only trials with P<1 were considered.

grouping	variable	full model	final model	null model

subject	intercept	0.01	0.01	0.01
	probability of the risky option	0.51	0.50	1.93
	value of the risky option	0.01	0.01	0.31
	experiment 1	0.30	0.29	1.06
	experiment 2	0.91	0.91	1.17
session within subject	intercept	0.14	0.04	0.01
	probability of the risky option	0.51	0.48	0.52
	value of the risky option	0.04	0.04	0.08
	experiment 1	0.40	0.42	0.30
	experiment 2	0.65	0.66	0.66

Table S9. Estimates of the full, final and null model investigating the impact of economic, specific and experimental parameters on risky choices in Experiment 1 and 2 combined. The table contains parameter estimates +/- s.e. for the final model. For categorical predictors (species, session, and side of the safe cup), the tested levels are indicated in parentheses. Only trials with P<1 were considered.

	full model	final model	null model
intercept	- 2.72 +/- 0.36	- 1.53 +/- 0.32	- 2.80 +/- 0.34
species (Orang-utan)	- 0.25 +/- 0.26		- 0.24 +/- 0.26
side of the safe cup (left of the subject)	- 0.70 +/- 0.12		-0.70 +/- 0.12
risky probability	2.08 +/- 0.57		2.27 +/- 0.49
risky value	0.45 +/- 0.05		0.45 +/- 0.05
experimental design (Experiment 2)	2.63 +/- 0.66		2.85 +/- 0.55
experimental design (Experiment 2) : risky value	- 0.26 +/- 0.06		- 0.25 +/- 0.06
experimental design	0.60 +/- 0.98		

(Experiment 2) : risky probability			

Table S10. Post-hoc tests investigating the impact of the experimental design on the level of risky choice. The table indicates: the trend estimates for the predictor: value of the risky option, and trend comparison for both experiments, as well as the estimated marginal means for both experiments and all values of the risky option, and the EMMs comparisons for both experiments.

	estimate	SE	z-ratio of the trend estimate or the marginal means comparison 1-2	p-value of the trend estimate or the marginal means comparison 1-2
Trend estimate for the predictor : value of the risky option (Experiment 1)	0.10	0.01	5.5	<.001
Trend estimate for the predictor value of the risky option (Experiment 2)	0.03	0.01		
EMMS (V=2, Experiment 1)	0.19	0.03	-5.31	<.001
EMMS (V=2, Experiment 2)	0.70	0.08		
EMMS (V=4, Experiment 1)	0.36	0.04	-4.6	<.001
EMMS (V=4, Experiment 2)	0.77	0.06		
EMMS (V=6, Experiment 1)	0.58	0.04	-3.11	<.005
EMMS (V=6, Experiment 2)	0.83	0.05		

Movie S1 (.mov format, available at 10.5281/zenodo.4710578). One trial of Experiment 1. The safe (yellow) cup is at the right of the subject, and the (pink) risky cup at the left.

Movie S2 (.mov format, available at 10.5281/zenodo.4710578). One trial of Experiment 2. The safe (blue) cup is at the right of the subject, and the (orange) risky cups at the left.
