# Supplementary material for: Rationality and cognitive bias in captive gorillas’ and orang-utans’ economic decision-making
Source: PLoS One. 2022 Dec 14;17(12):e0278150. doi: 10.1371/journal.pone.0278150 (PMC9749992; doi:10.1371/journal.pone.0278150)
Supplement: S2 File — (DOCX) [file pone.0278150.s002.docx]

S2 File. The two videos are available at this address: <https://zenodo.org/record/4710578#.Y5Bhax3jKXQ>.

Movie S1 : "Experiment_1.mov" and Movie S2 : "Expriment_2.mov".
