# Supplementary material for: Rationality and cognitive bias in captive gorillas’ and orang-utans’ economic decision-making
Source: PLoS One. 2022 Dec 14;17(12):e0278150. doi: 10.1371/journal.pone.0278150 (PMC9749992; doi:10.1371/journal.pone.0278150)
Supplement: Attachment — Submitted filename: Review_PLOS_Lacombe et al.pdf [file pone.0278150.s005.pdf]

Rationality and cognitive bias in captive gorillas' and orang-utans' economic decision-making

Overall, the manuscript is clearer now. However, I still find inconsistencies in how the statistical analysis are described and the results reported. I also have a few other comments in other manuscript sections that should be addressed before the final acceptance.

Introduction

L 135. The authors stated in ref. 39 that bonobos chose the risky option in 87.5% of trials. The numbers do not seem to coincide with figure 2 of ref. 39 unless the authors are averaging between small and medium sizes. Is this the case? Please clarify.

Methods

L 274. Table 1 seems to miss a minus value "(-)" after EV 1.

Statistical analysis

L 341: "we reached a model with interpretable term**s**".

L 356: I believe the added predictor is a sixth rather than a third predictor in the model. Experiment 2 analysis contains the same 5 predictors of Experiment 1 (risk probability, risk value, specie, session and side of the safe cup) + the addition of type of reward.

L 360: There seems to be an interaction between the position of the cup and species, as indicated in the results and in Table 5. This information is missing here, together with the fact that species was also a predictor.

L 372: I am confused because the null models included the species variable. To my understanding, that means the authors did not analyze the effect of species statistically (a full model including species against a null model without the variable) in any of their models, and therefore species differences should not be reported.

Results

General comment: The results are more precise than in the previous version of the manuscript, but I still have considerations about how they are reported. The authors constantly report main effects even when interactions between those main effects are significant. This is especially salient in Experiment 1. Probability to win, value of the risky option and session are all significant. Similarly, the interactions between session * probability and session * value are also significant. The authors should only report the interaction' effects and plot the results accordingly. In other words, the predictors' effect only makes sense in interaction with each other, not on their own anymore.

L 397: Please report more information on the significant two-way interaction. In which direction did they modify their choices?

Table 3: The inclusion of tables is helpful, but I would suggest the authors report more information regarding degrees of freedom, confidence intervals (values that are already calculated since they are plotted), and the model's estimates.

L 431: "..we found that the ~~the~~ session"

L 433: I would inform readers about the interaction between value and session. Also, I would suggest first reporting the effect of session in L 432 (that subjects increase their risk tendency over the experiment sessions) and then the non-significant effect of the interaction, clarifying that the apes did not learn the probabilities to win over repeated testing.

L 465: Same general comment I made earlier. If an interaction between the position of the cup and species is significant, the position of the cup on the trolley per se adds no value to the interpretation of the results.

L 483: The significant interaction between the type of experiment and the value of the risky option is not reported in table 6. In addition, what do the authors exactly mean by "subjects were more attentive to the value of the risky option in exp 1 than 2". It is unclear that attentive means that apes chose the risky option less in exp 1 compared to exp 2, as shown in figure 4A.

L 488; To clarify, trials with P = 1 refer to any trial with just two cups regardless of the number of rewards in the risky cup, right?

Discussion

L 525: I would add that controlled for expected gains means when EV = 2.

L 592: References missing for those experiments from experience providing partial feedback.

L 606: I would include the analysis in the manuscript. The authors reported the resulting plots to both reviewers in the previous review round. I think they should report the results of the analysis and the plots, at least in the supplementary materials, with a reference in the results section.

L 686: I would say that there were no *apparent* differences between species since the authors previously report an almost significant effect of species (p = 0.052) in Experiment 1 (although the authors may not require reporting species differences if their effect was controlled for and thus not statistically analyzed).
