# Supplementary material for: Rationality and cognitive bias in captive gorillas’ and orang-utans’ economic decision-making
Source: PLoS One. 2022 Dec 14;17(12):e0278150. doi: 10.1371/journal.pone.0278150 (PMC9749992; doi:10.1371/journal.pone.0278150)
Supplement: Attachment — Submitted filename: Review PLOS ONE 2021.docx [file pone.0278150.s003.docx]

The article aims to investigate how gorillas and orangutans behave in two risk-assessment tasks. In experiment 1, the apes experienced a single-choice scenario where they had to decide between a cup containing a secure reward and a cup that may contain a high-value reward. Importantly, they did not know beforehand the quantity of reward at stake and whether a reward would be present or not at any given trial—although they might learn the probability that the reward was present after some experience. In experiment 2, apes experienced a multiple-choice scenario where again they had to decide between a cup containing a secure reward and a choice between a maximum of four risk options—only one being baited with a reward. Importantly, in the second study, the apes knew the risky reward at stakes and that it was always baited inside one of the cups. They found that both species seem to act rationally, increasing their choices towards the risk option when the expected value for the risk option is higher, when the probability to obtain it is higher and when its value is higher. However, they find differences between experiments, with both species being more risk-prone in experiment 2 (in those trials in which the expected value of the secure and the risky choice was the same) and differences between species, with gorillas being more risk-prone than orangutans in experiment 1.

The studies are well-conducted, and the statistical analysis is correct. In the discussion, the authors do a good job describing potential hypotheses to explain their results. However, I would like the authors to clarify some points before final acceptance.

Uncertainty vs. risk tasks

My main comment concerns the difference between uncertainty/ambiguity and risk scenarios. The authors interpret their two studies as tasks suitable to test risk preferences. However, in their discussion, they comment that during the single-cup experiment 1, apes learn the probabilities by experience (as opposed to experiment 2, in which the exact probabilities are described since apes can observe the risky reward and the number of cups). While I agree with the distinction, I am surprised that the authors do not discuss differences in terms of ambiguity/risk. One possibility is that in experiment 1, it is harder to learn the probability of appearance despite the experience. Although the study reports that a rise in the probability to win had an increasing effect on apes risk choices, one could imagine that apes were often deciding under conditions of ambiguity (especially at the beginning of experiment 1 session), and that could partially explain differences between experiments 1 and 2 overall preferences towards risk. If that were the case, the results would also align with previous studies by Rosati & Hare, 2011 and Haux et al., 2021.

Introduction

L 99 and 105: I am not sure if the ref. 26 interpretation is accurate. The chimpanzees were risk-prone in that study. Furthermore, risk options always provided rewards (either less or more grapes than the secure option).

L 132: I would stick with rewards instead of awards.

L 180: Check if the hypothesis are correct. The authors mention that they expect risk proneness in both tasks, but in L 99, they argue that primates are mostly risk-averse. I guess the authors expect more risk aversion or neutrality in experiment 1, as they have found.

Training:

In general, some details are missing. Which were the quantities involved in the training? Did they need to reach a rate of 80% correct over how many trials?

Also, what was the purpose of the hidden condition, to demonstrate that they can remember the quantities through the transparent saucer before they are covered? Did they then also need to reach at least an 80% of success in those trials? Please specify this in the manuscript.

Experiment 1:

L 260: I wonder if the authors could analyze the session effect in experiment 1 as they did in experiment 2 (instead of having session as a random effect). A session effect could tell us whether apes were learning the EV across time—increasing or decreasing their risk choices depending on the probability of obtaining the reward (P) and its value (V). The last comment relates to my previous one on the difference between ambiguity/risk choices.
It might also help to specify whether the presentation order varied between the four sessions. For instance, if the probability of food present in the risk choice was 0.5, was food present in the risk option every second trial, or was the presentation randomized as long as there would be a total of 5 trials with and without food? If trial presentation order was blocked within sessions, then ambiguity was reduced since the four sessions would be exactly the same.

Statistical analysis:

The description of the models is very clear except for the fixed-effects part. The authors describe the random effects and the additional fixed effects for experiment 2, but it is unclear which are the shared fixed effects between studies (e.g., 318). It is only apparent in the result section (e.g., the value of the risky option).

L 323: another instead of an other.

Results:

I would remind the readers that the probability of the risky option refers to P in the table, that the value of the risky option refers to V and that the expected value refers to EV =P*V.

Discussion:

L 444: Ref 26 leads to risk aversion only in bonobos.

L 487: Close parenthesis after reference 43.

L 492: either "described" or 'described'.

L 493 to 495: I find these sentences slightly unclear (e.g., "with feedback the chosen option").

L 512: It might be interesting to report some descriptives of the pilot regarding the latencies discussed.

L 563: I understand the logic in the possible distinction between the risk assessment task in the domain of gains (experiment 1) and the domain of losses (experiment 2). However, how do we know whether apes took the risk choice as the reference in experiment 2? Perhaps the authors could cite some prospect theory literature showing whether better options are usually taken as reference points?
Also, the authors mention that, given that the risk option is the reference, choosing it leads to the loss of all the reference quantity—instead of a proportion of it when choosing the safe option. However, that might not be true for all trial constellations since sometimes the risk option is equally probable, and they can also obtain it from time to time. In other words, they do not permanently lose all the “reference” quantity. Otherwise, they would always take the safe option.

L 596: close parenthesis after Experiment 2.

L 610: I would suggest making the potential role of single cup design more explicit.
