# Supplementary material for: Rationality and cognitive bias in captive gorillas’ and orang-utans’ economic decision-making
Source: PLoS One. 2022 Dec 14;17(12):e0278150. doi: 10.1371/journal.pone.0278150 (PMC9749992; doi:10.1371/journal.pone.0278150)
Supplement: Attachment — Submitted filename: review_13dec_final.docx [file pone.0278150.s004.docx]

**Detailed answers to the editor’s and the reviewers’ comments**

**Editor:**

correctly citing the literature on previous risk preference studies in nonhuman primates (as evidenced by both reviewers, not all nonhuman primate groups tested so far were risk averse; for instance, capuchins tested by the De Petrillo et al 2015 – your reference # 22 – were risk prone when the risky and the safe options had the same EV);

Modification of the manuscript : lines 95 to 119.

better address in the Discussion the limits of your study in terms of the small sample size, which does not allow to properly evaluate age and sex effects;

We modified the manuscript following this suggestion (the effect of age and sex are no longer analysed) and we addressed the limitation of our conclusions (lines 677 and 706).

take into account all the methodological and statistical concerns raised by Reviewer

Modification of the manuscript : lines 326 to 369.

253-55 the graphical solution doesn’t seem ideal, maybe it would be better to use plain text with three different symbols beside each number for indicating whether the EV of the risky option was higher, equal or lower than that of the safe option

Modification of the manuscript : table 1 and 2.

l 286 shouldn’t it be EV=2?

Modification of the manuscript : line 305.

L 304 “to check for within learning effects”: something seems to be missing here

Modification of the manuscript : line 322.

L 327 “paired t-test”: didn’t you use single-sample t tests to assess whether risky choices significantly differed from the chance level?

Indeed, this was an error in the manuscript. Modification of the manuscript : line 370.

L 361: ‘Multiple cup’ design

Modification of the manuscript : line 415.

L 377 “Results of Experiment 2” is repeated twice

Modification of the manuscript : line 437.

L 432 perhaps “had” rather than “formed” here?

Modification of the manuscript : line 513.

L 495 “with feedback from the chosen option”

Modification of the manuscript : line 579.

L 560 “do not gain”

Modification of the manuscript : line 644.

L 562 please delete “that”

Modification of the manuscript : line 646.

L 566 “a” rather than “an”

Modification of the manuscript : line 650.

L 596 “species”

Modification of the manuscript : line 677.

I suggest to delete, or extensively rephrase, the paragraph on ll 596-615, as it does not point out at the feeding ecology differences between gorillas and orangutans (and, indeed, a thorough discussion on the species differences is not warranted given the small sample sizes) and I am not sure whether it constructively contributes to the Discussion.

The aim of this paragraph was not to discuss species differences but to discuss the relevance of each protocol in the study of economic strategy in great apes. Our point was that, in order to investigate great apes’ economic decision-making, one should confront the subjects with similar choices as the ones they would face in the wild. The conclusion is that, given the ecology of gorillas and orang-utans, a mixed of both designs would have more ecological relevance.

L 630 please delete “of” (repeated twice)

Modification of the manuscript : line 712.

L 636 “on” rather than “towards”?

Modification of the manuscript : line 718.

LL 672-3 “respectively”

Modification of the manuscript : lines 761, 762.

Fig 2 “(B)” is missing

Modification of the manuscript : Fig 2.

Supplementary material: is it the spelling of the video location correct? Shouldn’t it be “zenodo” rather than “zenedo”?

Modification of the manuscript :lines 769, 772.

**Reviewer #1:**

Premise isn’t solid. The authors state “Generally, most primates and non-primates are risk-averse” (l 99). This is contradictory to what they say prior to this (cognitive biases etc, showing context specificity of risk preferences in humans) and, importantly, this generic statement is also not adequate for nonhuman primates as a group. For example, several studies found chimpanzees to be risk-seeking (sometimes in comparison to bonobos) with procedures similar to the two-cup method (e.g., Heilbronner et al., 2008; Rosati & Hare, 2011, 2012, 2013) and in another study the authors cite already, different apes and monkeys were not throughout risk-avoidant (Broihanne et al., 2018). Since the introduction, in its current form, relies on this premise to identify the knowledge gap that the current study aims to fill, it doesn’t seem fit. There are also very different procedures, e.g. eye gaze instead of pointing to a cup, that find nonhuman primates to be risk-seeking sometimes.
In sum, I think the introduction needs some work to adequately reflect the state of current literature and integrate the current study coherently into this picture.
Some examples of relevant literature re risk-proneness in chimpanzees and eye gaze method and two recent studies showing chimps to be more risk-averse, one using a two-cup setup and one using a more complicated apparatus

Modification of the manuscript : lines 95 to 119.

I felt that, in general, the results and conclusions should be phrased more carefully, considering the small sample size. Don’t get me wrong, small sample size is not a reason not to publish a study. However, we all have the responsibility to point out obvious limitations in the scope of our analysis and conclusions. Sample sizes of n = 5 and n = 3 neither warrant analysis of individual factors such as sex per species nor statements such as “Our results are in stark contrast to the literature, …” (L 438-440), especially considering that the referred papers also had relatively small sample sizes. The current results add an interesting piece to the puzzle, but I wouldn’t go as far as claiming they show a huge discrepancy, yet. In this respect, I was also wondering if all the individuals showed similar patterns or behaved very differently with perhaps one individual driving the effect? (I raise this point further below in reference to result presentation).

We modified the manuscript following this suggestion (the effect of age and sex are no longer analysed) and we addressed the limitation of our conclusions (lines 677 and 706). For individual data, see below.

Methods & statistics & results: the sections need to be substantially fleshed out. Important pieces of information are missing, the rationale for the statistical approach is not sufficiently explained and model descriptions & results need more detailed information.

Modification of the manuscript : lines 326 to 369.

Also, what is a “discontent analysis”? – this is mentioned in the description of the data files but has no match in the manuscript.

We removed that analysis of our study.

The information provided in the manuscript is not sufficient to understand which models were run, which predictors were included**,** how these predictors were treated (as categorical or continuous) and which model set out to answer which particular question.
For example, l. 309-311 – what does “risky option” refer to (the probability of the option or its size?) and which individual characteristics were assessed?
L. 319-320 – is it correct that ‘session’ entered the model as both fixed and random effect? Also, in none of the models, “protocol” seems to be included as a predictor, but wasn’t this the main goal of the study?

L 318-319: What are the fixed effects? What do you mean with “adding a significant interaction”? If the model is specified as including the interaction term as a predictor, then running the model and comparing it to respective null model will reveal which terms explain considerable parts of the variance. But you can’t only add a predictor term after you found it somehow to be significant – maybe this is a misunderstanding due to the way the sentence is phrased in the current version of the manuscript. But similar wording is in l 335-336, so I wonder what exactly were the steps that the authors performed during their analysis?
Currently, the analysis section reads like a long list of GLMMs and t-tests, but it’s difficult to follow which question each of them answers and which part of the results section refers to which of these models/t-tests. Why do you run additional t-tests when you already test for risk-preference with the GLMMs? In addition, from the information that is provided, random slopes are missing from the GLMMs. However, these are important to model effects of fixed effects among levels of random effects and to avoid inflated Type 1 error rates.I would like to see some more information on the paired-samples t-Test that was performed to test for side bias (as stated in L 334) and provided the results reported in L 383-390 – I am struggling to see how a t-Test can account simultaneously for species differences, different number of possible positions to choose depending on number of presented cups, and side of safe reward. Or were several t-Tests run on the same data to test for these effects? In this case, which method was used to account for multiple testing, and was there a reason why these aspects were not considered as predictor terms in the GLMM in the first place?

Modification of the manuscript : lines 326 to 369.

I didn’t find information about inter-rater reliability.

Modification of the manuscript : lines 326 to 328.

**Results:**

It was difficult to follow the results description and judge its appropriateness, given the missing information as outlined above**.** A table specifying the model output would help a lot, specifying for all predictor terms the respective estimates, standard errors, confidence intervals, and test results (likelihood ratio test, degrees of freedom, p-value). And information if the model was overall different from a model excluding the predictor terms of interest; consider including effect size in result reporting, as well.

Modification of the manuscript : tables 3, 4, 5 and 6. The result sections were also re-written following your suggestions (lines 382 to 397, 416 to 432, 452 to 455, and 473 to 475).

L 355: is this 45.7% for Gorillas or Orangutans? And the other species?

Modification of the manuscript : lines 400 to 403.

I don’t think assessing effects of age and sex statistically is warranted, given the samples only have 1 male per species, only 1 adult for the orangs and only 1 juvenile for the Gorillas.

We modified the manuscript following this suggestion (the effect of age and sex are no longer analysed).

L 400-406: which data and which model corresponds to these results?

Modification of the manuscript : table 6.

Figures: It would be nice to see the individual data, especially because there are so few subjects.

**Individual data for Experiment 1**

**Individual data for Experiment 2 (low-level reward)**

X-axis labelling should be adapted so it shows the possible values for the different figures (e.g., A only allows values of 2,4,6 and 7 but shows also integers in between, whereas C allows multiple values but only shows 1,2,3, and 4). Confidence intervals overlap for different lines and hide the CI of the other line (species or reward value); applying a jitter function might help.

Modification of the manuscript : figures 2, 3, 4.

Caption Fig.2: missing (B)**.** Delete “for low valued reward” – its not necessary because no high value reward condition was presented.

Modification of the manuscript : figure 2.

Caption Fig.3: delete “Results of Experiment 2”.

Modification of the manuscript : figure 3.

**Methods:**

L 189-192: please, provide more details about subjects here (at the very least that only 1 male was in each group) and refer to supplementary table.

Modification of the manuscript : lines 195 to 200.

In the discussion, a pilot study is mentioned; which is contradictory to subjects being completely naïve to testing prior to procedure of Exp.1. Was the pilot part of this study and what was it about? Is it of relevance to the subjects’ testing history?

The wording “pilot study” was confusing. We just analysed the response times in both experiments and received the following results (see figure below).

L 215-28: which quantities were used? What does “success rate of >=80%” mean? Did they have to reach this criterion for different quantity discriminations, or overall, or in a specified number of consecutive test sessions? How many trials did it take the individuals to reach this criterion? These informations are important to get an idea how stable the 80% performance rate was or whether it might have been a lucky accident.

Modification of the manuscript : lines 234 to 247.

L224-226: Did the subjects first see where food was placed and then pointed to the now hidden rewards, or did they have to learn to associate a cup colour with a specific content by sampling information?

Modification of the manuscript : lines 234 to 247.

L232-242: did subjects see in advance which quantity might be hidden under the risky cup?

No, they did not.

Why did you use two experimenters?

To conduct a double-blind procedure.

On the videos it looks like E2 is watching E1 hiding the food, was this always the case?

No, this was not the case: E2 stayed close to E1 but was not looking at where the food was hidden.

L260-265: How was number trials decided? (for example, based on a simulation to find out necessary number of trials to find an effect, if there is one; or based on previous literature).

In Heilbronner et al (2008), subjects were tested in an analogous design of Experiment 1 and the learning effect was analyzed: subjects risk-preference were stable between the 3 blocks of 3 sessions (10 trials per session). 30 trials were then sufficient to establish risk-preference. In Hayden et al (2008), and Long et al (2009) similar tasks (risk-assessment in a design similar to the “single-cup” design) were conducted using blocks of 25 to 40 trials per condition.

As we conducted 20 trials per day, we performed 40 trials (2 sessions of 10 trials) for each PxV combination.

Design table: in a 10-trial session, how were the different win probabilities realized? How many wins and losses were presented per session per condition? It doesn’t add up for me. For example, .33 -> did you bait 3 or 4 wins within 10 trial session?

For P=0.3, we assigned 1 (baited cup) in every 3 trials, ex : 0 1 0 0 0 1 0 1 0 1 0 0 0 1 0 1 0 0 1 (0 0). The last two trials were not performed as we conducted 20 trials per day.

For P=0.25, we assigned 1 (baited cup) in every 4 trials, ex : 0 1 0 0 0 0 1 0 etc.

For P=5, we assigned 1 (baited cup) in every 2 trials, ex : 0 1 0 1 1 0 0 11 0 0 1 etc.

Exp. 2: did all subjects of Exp1. Participate in Exp. 2? Was there a break between the experiments?

No, one subject (female adult gorilla: Adira) dropped the experiment between E1 and E2, and one subject (male adult orang-utan: Bagus) only did E2+ (E2 with high-reward food), see table S1.These two subjects were removed from the whole experiment. Two subjects that completed E1 only completed E2- (E2 with low-reward food). They were included in the results of E1 and E2- and in the comparison of E1 and E2- (as both experiments used low-reward food), but not in the results of E2+.

There was a break between the experiments of one week.

Did they receive a familiarisation training with the new procedure to learn that even when there are more cups than previously, they still only get to pick 1 and not more?

No, but they did not try to pick several cups.

Why was the order of food type not counterbalanced between individuals?

This would have been preferable, but we obtained the authorisation from the veterinary to use high-valued food after we started the experiment. For one subject (Bagus), we started E2 with high-valued food, but he did not performed E2 with low-valued food so he was removed from the experiment.

Why is there an additional value (7 rewards) for Exp 2?

We wanted to increase the number of conditions where the expected value of the risky option was generally higher than the expected value of the safe option (without the value of 7 rewards we would have tested only one such condition where P<1 and EV > 2). This was important especially for Exp 2 as the design of Exp 1 has been studied and tested on several species before, and previous work showed that subjects understood the economic nature of the task, while Exp 2 has only been performed once (in the same design that we run it) and, prior to our study, it was still unclear weather subjects understood the task.

We were allowed a maximum value of 7 rewards per trial by the zoo veterinarian.

L297-299: I don’t understand this reasoning. Why was it necessary to prevent subjects from learning about the win probability? Isn’t it necessary for the subject to understand the probabilities to make an informed decision about whether to play it safe or not?

In E2 we did not want subjects to learn the probabilities to win, but to understand and infer probabilities without training from the number of risky cups. This is why the number of risky cups was semi-randomised within a session.

L301-303: this sentence is confusing – how is each reward amount, all numbers of cups be tested 5 times within a session that only contains 10 trials?

Each reward amount is tested during 2 non-consecutive sessions of 10 trials: among these 20 trials, each cup number (1, 2, 3 or 4) is tested 5 times.

L297-305: Its very hard to follow the description of number of trials, sessions, and experiment repetitions. E.g. l303-> could you just say that you presented 16 sessions per reward type, i.e. 32 sessions in total? It might help to present an example of one of the orders (either in the main text or as part of the supplementary material).

For one subject and one type of reward:

We performed two runs of the experiment to check for learning effects. For each run of Exp 2, we conducted 16 sessions. During each sessions we tested one reward quantity, and varied the number of risky cups between trials. Throughout the 16 sessions of one run, each PxV combination was tested 5 times (for instance in the table the grey cells corresponds to the 5 occurrences of the V=2, P=0.25 combination for the first run).

**Run 1**

**Reward :	Number of cups :**

| N=2 | 1 | 3 | 2 | 4 | 1 | 3 | 2 | 4 | 1 | 3 |
| --- | --- | --- | --- | --- | --- | --- | --- | --- | --- | --- |
| N=7 | 3 | 2 | 4 | 1 | 3 | 2 | 4 | 1 | 4 | 2 |
| N=4 | 2 | 3 | 1 | 4 | 2 | 4 | 3 | 1 | 4 | 2 |
| N=6 | 3 | 1 | 4 | 1 | 3 | 2 | 1 | 4 | 2 | 3 |
| N=6 | 4 | 2 | 3 | 2 | 1 | 4 | 2 | 3 | 1 | 4 |
| N=4 | 2 | 4 | 3 | 3 | 1 | 2 | 4 | 1 | 3 | 1 |
| N=2 | 3 | 1 | 2 | 4 | 4 | 1 | 2 | 3 | 4 | 2 |
| N=7 | 4 | 3 | 1 | 2 | 4 | 3 | 1 | 1 | 3 | 2 |

**Run 2**

| N=7 | 3 | 1 | 2 | 4 | 4 | 1 | 2 | 3 | 4 | 2 |
| --- | --- | --- | --- | --- | --- | --- | --- | --- | --- | --- |
| N=6 | 2 | 4 | 3 | 3 | 1 | 2 | 4 | 1 | 3 | 1 |
| N=4 | 4 | 2 | 3 | 2 | 1 | 4 | 2 | 3 | 1 | 4 |
| N=6 | 2 | 3 | 1 | 4 | 2 | 4 | 3 | 1 | 4 | 2 |
| N=4 | 3 | 1 | 4 | 1 | 3 | 2 | 1 | 4 | 2 | 3 |
| N=2 | 4 | 3 | 1 | 2 | 4 | 3 | 1 | 1 | 3 | 2 |
| N=7 | 1 | 3 | 2 | 4 | 1 | 3 | 2 | 4 | 1 | 3 |
| N=2 | 3 | 2 | 4 | 1 | 3 | 2 | 4 | 1 | 4 | 2 |

Modification of the manuscript : figure S3.

I was missing an explanation why not all expected values tested in Exp.1. And why not both high and low quality food types? Seems to make it difficult to directly compare Exp. 1 and Exp.2.

It would have been preferable to test all values in Exp.1 and to test both quality food types. Unfortunately, the experiment was already quite complex and very long to run (2.5 years), therefore we could not test the combinations of every parameters (EV, type of food) in Experiment 1 (testing one condition takes 4 times longer in Experiment 1 than in Experiment 2).

Discussion

L 443-444: “an experimental design usually leading to risk-aversion [26]”. But Ref 26 found risk aversion in bonbos and risk proneness in chimps and thus doesn’t fit the statement very well

Modification of the manuscript : lines 522-523.

L 452-454: “Finally, [28] could not reject that subjects understood the task and properly inferred the probabilities to win without experience. In this study, we fixed these experimental issues” -> explanation required how exactly the current study fixed the issue.

We modified the experimental design (counterbalancing the sides of safe and risky options, removing the refreshers trials, adding of a condition where the risky option is as large as to the safe option, using a low-level reward,...) which resulted in lower levels of risky choices, as expected, and allows to address [28] concern that subject did not understand the task, as we showed that they responded rationality to variations in probability or potential gain. This is mentioned in detail in the introduction of the manuscript, lines 144 to 155, and 176 to 180.

L 456-457: On the videos provided, it looks like the baiting procedure is well in sight of Experimenter 2. Was this always the case?

No, this was not the case: E2 stayed close to E1 but was not looking at where the food was hidden.

L 457: “levels of risky choices” -> does this refer to the size of the risky option?

No, it refers to the percentage of risky choices (this expression was used before in the result section, but can be modified if not clear).

L 458-459: “probability to win to verify that subjects based their strategy on the value of that probability and were thus able to compute it” -> its not fully clear to which of the listed results this is referring.

Modification of the manuscript : lines 537 to 544.

L 512. What pilot study? It is mentioned the first time here, should the reader know about it already at this point?

The wording “pilot study” was incorrect, we are referring to the analysis of response times in both experiments: we measured subjects' response times for every trial of Experiment 1 and 2 to test whether there was a relationship between RT and experimental design. As the result was inconclusive (longer response times in Experiment 2, which could be interpreted as due to the fact that subjects had more cups to choose from).

The “lateral hypothesis”. The position bias is interesting, and also that the two species apparently had different kind of bias. However, what does this tell us about the risky choice results? Are they meaningful at all when subjects had a clear position bias? I think this point deserves mention and being discussed.

We addressed this point in the discussion (lines 244 to 247, and 255 to 258) : even though subjects had a clear position bias, this doesn't affect risk-assessment results in gorillas (as the side of the safe option was counterbalanced between trials). For orang-utans, the lateral bias apparently interacts with subjects' choice between the safe and risky option, but as the total number of risky cups is a good predictor of levels of risky choice, this lateral bias is not the only predictors of subjects' choices, as they are making rational economic decisions.

**Reviewer #2:** 

Uncertainty vs. risk tasks
My main comment concerns the difference between uncertainty/ambiguity and risk scenarios. The authors interpret their two studies as tasks suitable to test risk preferences. However, in their discussion, they comment that during the single-cup experiment 1, apes learn the probabilities by experience (as opposed to experiment 2, in which the exact probabilities are described since apes can observe the risky reward and the number of cups). While I agree with the distinction, I am surprised that the authors do not discuss differences in terms of ambiguity/risk. One possibility is that in experiment 1, it is harder to learn the probability of appearance despite the experience. Although the study reports that a rise in the probability to win had an increasing effect on apes risk choices, one could imagine that apes were often deciding under conditions of ambiguity (especially at the beginning of experiment 1 session), and that could partially explain differences between experiments 1 and 2 overall preferences towards risk. If that were the case, the results would also align with previous studies by Rosati & Hare, 2011 and Haux et al., 2021.

This figure shows the level of risky choices in E1 for the 4 sessions of each PxV combination. We cannot explain the difference between E1 and E2 by the sole hypothesis that subjects make their choices under ambiguity in E1, because, as subjects learn the probability (and the quantity of reward) throughout the 4 sessions of 10 trials for each PxV condition of E1, the level of risky choices does not drastically increase (on the contrary, the level of risky choices tends to decrease).

L 99 and 105: I am not sure if the ref. 26 interpretation is accurate. The chimpanzees were risk-prone in that study. Furthermore, risk options always provided rewards (either less or more grapes than the secure option).

Modification of the manuscript : lines 95 to 119.

L 132: I would stick with rewards instead of awards.

Modification of the manuscript : line 146.

L 180: Check if the hypothesis are correct. The authors mention that they expect risk proneness in both tasks, but in L 99, they argue that primates are mostly risk-averse. I guess the authors expect more risk aversion or neutrality in experiment 1, as they have found.

Modification of the manuscript : lines 186, 187.

*Training:*

In general, some details are missing. Which were the quantities involved in the training? Did they need to reach a rate of 80% correct over how many trials?
Also, what was the purpose of the hidden condition, to demonstrate that they can remember the quantities through the transparent saucer before they are covered? Did they then also need to reach at least an 80% of success in those trials? Please specify this in the manuscript.

Modification of the manuscript : lines 234 to 247.

*Experiment 1:*

L 260: I wonder if the authors could analyze the session effect in experiment 1 as they did in experiment 2 (instead of having session as a random effect). A session effect could tell us whether apes were learning the EV across time—increasing or decreasing their risk choices depending on the probability of obtaining the reward (P) and its value (V). The last comment relates to my previous one on the difference between ambiguity/risk choices.

Modification of the manuscript : lines 388 to 393.

It might also help to specify whether the presentation order varied between the four sessions. For instance, if the probability of food present in the risk choice was 0.5, was food present in the risk option every second trial, or was the presentation randomized as long as there would be a total of 5 trials with and without food? If trial presentation order was blocked within sessions, then ambiguity was reduced since the four sessions would be exactly the same.

For P=0.3, we assigned 1 (baited cup) in every 3 trials, ex : 0 1 0 0 0 1 0 1 0 1 0 0 0 1 0 1 0 0 1 (0 0). The last two trials were not performed as we conducted 20 trials per day.

For P=0.25, we assigned 1 (baited cup) in every 4 trials, ex : 0 1 0 0 0 0 1 0 etc.

For P=5, we assigned 1 (baited cup) in every 2 trials, ex : 0 1 0 1 1 0 0 11 0 0 1 etc.

Ambiguity was then reduced as we limited the variability of the occurrence of baiting and baited the risky cup with a frequency that was highly similar to the probability.

*Statistical analysis:*

The description of the models is very clear except for the fixed-effects part. The authors describe the random effects and the additional fixed effects for experiment 2, but it is unclear which are the shared fixed effects between studies (e.g., 318). It is only apparent in the result section (e.g., the value of the risky option).

Modification of the manuscript : lines 326 to 369.

*Results*:

I would remind the readers that the probability of the risky option refers to P in the table, that the value of the risky option refers to V and that the expected value refers to EV =P*V.

Modification of the manuscript : lines 382 to 384, and 416 to 418.

*Discussion*:

L 444: Ref 26 leads to risk aversion only in bonobos.

Modification of the manuscript : lines 522, 523.

L 487: Close parenthesis after reference 43.

Modification of the manuscript : line 572.

L 492: either "described" or 'described'.

Modification of the manuscript : line 577.

L 493 to 495: I find these sentences slightly unclear (e.g., "with feedback the chosen option").

Modification of the manuscript : line 579.

L 512: It might be interesting to report some descriptives of the pilot regarding the latencies discussed.

We analysed the response times in both experiments and received the following results (see figure below).

L 563: I understand the logic in the possible distinction between the risk assessment task in the domain of gains (experiment 1) and the domain of losses (experiment 2). However, how do we know whether apes took the risk choice as the reference in experiment 2? Perhaps the authors could cite some prospect theory literature showing whether better options are usually taken as reference points?

We don't know whether the apes took the risk choice as the reference, but if they did, that would explain our result. We just brought up that hypothesis, but it would indeed have to be tested.

Also, the authors mention that, given that the risk option is the reference, choosing it leads to the loss of all the reference quantity—instead of a proportion of it when choosing the safe option. However, that might not be true for all trial constellations since sometimes the risk option is equally probable, and they can also obtain it from time to time. In other words, they do not permanently lose all the “reference” quantity. Otherwise, they would always take the safe option.

Indeed, the sentence was incorrect.

L 610: I would suggest making the potential role of single cup design more explicit.

Modification of the manuscript : line 693.
