# Supplementary material for: Rationality and cognitive bias in captive gorillas’ and orang-utans’ economic decision-making
Source: PLoS One. 2022 Dec 14;17(12):e0278150. doi: 10.1371/journal.pone.0278150 (PMC9749992; doi:10.1371/journal.pone.0278150)
Supplement: Attachment — Submitted filename: letter-revision-6 avril.odt [file pone.0278150.s006.odt]

Cover letter and detailed answers to the editor’s and the reviewers’ comments

Ref.: Ms. No. PONE-D-21-29220R1 
Rationality and cognitive bias in captive gorillas and orang-utans economic decision-making
PLOS ONE


Dear Dr Elsa Addessi, Academic Editor of PLOS ONE,
We would like to express our appreciation for the reviewers' useful comments and contribution, especially concerning the statistical analysis. We implemented all changes as suggested by the editor and the reviewers. In the following we addressed all comments and questions in details.
Our best regards,

Pénélope Lacombe, Christoph Dahl.


Reviewer #1:

In brief, my main points concern the analysis. Particularly missing random slopes and additional t-tests. If the results hold when random slopes are included in the models, or if the authors can justify why they do not need a random slopes structure in their analysis (maybe I overlooked something in the design of the study?), then that’s great and I have no other major concerns.
Random slopes were added to our model and we compared the full model with random slopes to simpler model removing random slopes one by one in order to keep the most parsimonious one. See lines 339->344 and supplementary tables S2, S6 and S9.

L 339-340: “The significance of each predictor variable in explaining variation in rate of risky choices was tested with ANOVA” -> Could the authors clarify what they mean with this in the context of a full-null model comparison approach? In my understanding, when model comparison found that two models differ then the model output statistics tell about contribution of each predictor to explain variation and no additional ANOVAs are needed.
The phrasing we used was confusing. We did not performed an “ANOVA” but an analysis of deviance (type II Wald chisquare test) in order to test the significance of predictors. We removed the comparison to the null model from our analysis.

• L 335-343: to which analysis is this model referring? If this is meant as a general paragraph to report that assumption checks etc were done for each of the analyses, please clarify accordingly. The paragraph about full-null model comparisons should be moved up here.
Is is indeed a general paragraph We made it more clear (line 324).

L 345-354: description of the full model to analyze Exp.1. Just minor, but this was a bit difficult to understand, because on the one hand the authors count predictor variables (up to 5), but on the other hand they have more predictor terms in their full model, namely the additional two 2-way interactions. Maybe this could be phrased a bit differently, something like this: Our full model included x and y … as well as their interaction as fixed predictor terms of interest. To control for z and xx…, we also included these as fixed effects but kept them in the null model.
We made it more clear (lines 334 to 339)

L 373: Shouldn’t species be a predictor of interest, why was it kept in the null model? I understood a potential species difference was one of the main research questions.
Indeed species should not be kept in the null model. In any case, the comparison to the null model was removed (we tested the significance of predictors with analysis of deviance).

L 357-361: Is this part of the original confirmatory analysis plan to test the research question of risk preference? It sounds more like a position/side bias check. It would be helpful if this was clarified explicitly.
We made it more clear (line 362).

This reminds me of a methodological question: according to which logic were hidden reward positions determined? I assume this was somehow balanced such that each of the four possible risky positions held a risky cup equally often and a reward was hidden at each position equally often across the different number of risky cup trials? Or were risky cups always positioned immediately next to the safe cup with no empty positions when fewer than 4 risky cups were presented? It information would be useful for readers who want to use the paradigm and replicate the study.
Indeed, we balanced the position of the baited risky cup so that each risky cup help the reward equally often. We made it more clear (lines 290->291).

L 376-381: The point of modelling risk preference as a function of the above specified predictor variables is to account for the influence of these predictor variables. The described t-tests ignore these aspects. Assuming, for example, the t-test reveals risk neutrality, but the GLMM shows a clear effect of session (for example, apes are risk averse at the beginning and become increasingly risk-seeking throughout the study) – Then what have you gained with this t-test? It provides less and potentially misleading information. Therefore, I find the described t-tests inappropriate here.
Indeed, we removed that analysis from our manuscript. We tested risk-preference at the indifference point using post-hoc tests on our models (see lines 345->352, and result section).

I am still missing random slopes in the model structure. Random effects need to be considered when individuals provide repeated observations. But here, individuals contribute repeated observations under different repeated conditions (i.e., the different values & probabilities of risky option, different sessions in which these conditions are tested for every individual, etc), which should be modeled via appropriate random slopes. In this case, I think one would want to include random slopes of all predictor terms of interest as well as session within individual and of all predictor terms of interest within session. (See e.g. Barr et al, 2013, for why random slopes are important to draw reliable inference about fixed effects 10.1016/j.jml.2012.11.001)
Random slopes were added to our model and we compared the full model with random slopes to simpler model removing random slopes one by one in order to keep the most parsimonious one. See lines 339->344 and supplementary tables S2, S6 and S9.

In general, I would like to see the models run with random slope structure to see if the results hold; nonetheless, I have some comments on the current results.
Random slopes were added to our model and we compared the full model with random slopes to simpler model removing random slopes one by one in order to keep the most parsimonious one. See lines 339->344 and supplementary tables S2, S6 and S9.

L 390 – 398: The authors report about main effects of value of risky option, probability to win and session but then report the two interactions including these variables turned out significant. This means the main effects should not be interpreted because of limited use when they interact with other variables.
Indeed, we modified the manuscript and report the main effects but do not interpret it anymore.

L 400-404: I suppose these results are based on the additional t-tests not on the model comparison, because the authors report earlier that species remained a predictor in the null model. It would help if it was mentioned again to the reader to which analysis the respective results belong. (but see my general critique of the t-tests above)
We modified that analysis and do not perform t-test anymore (see lines 406-413).

L 481-486: If risky choice was affected by an interaction of type of experiment and value of the risky option, then the main effect of experimental design shouldn’t be interpreted (l 481-482).
We modified the manuscript and removed that interpretation.

L 485-487 this sentence doesn’t seem to reflect the model results and Fig. 4B. Model results show no interaction between experimental design and probability to win, and Fig. 4B depicts something that looks more like a main effect of experiment (with more risky choice in Exp. 2) with the exception of when p =1, to which individuals seem to be sensitive in Exp. 2 but not Exp. 1.
Indeed there was a mistake in the sentence, we modified the manuscript (line 471->474).

L 524-526: “in particular, if controlled for expected gains, subjects were generally risk-neutral in Experiment 1 and risk-prone in Experiment 2.” I don’t know what the authors mean by this and how they controlled for expected gains. 
We made it more clear (see line 515).

Is this referring to section “Performance in 'single cup' vs 'multiple cup' design”? If yes, please clarify whether main effect of experimental design can be interpreted despite interacting with risk probability. Also, why is expected gain a non-economic parameter?
Yes it is referring to that section. In our opinion, the effect of experimental design is multiple : 
there is an overall higher level of risky choices in Experiment 2 than in Experiment 1 (see Fig. 4 and post-hoc tests)
the experimental design affects subjects' perception of the P=1 condition (see Fig. 4)
the experimental design affects subjects' perception of the value of the risky option (analysis of deviance in table 6 and Fig. 4)
We did not mean than expected gain is a non-economic parameter. The wording was not clear, we were only referring to experimental design when we mentioned “non-economic parameters”.

L 543: For clarification that this paragraph refers specifically to Exp. 2, add “In this study, we addressed these experimental issues of the multiple cup design”
We made it more clear (line 533)

551: remove “did”
Done

628-630: “However we found no support due to the fact that both species exhibited positional biases (gorillas: lateral bias; orang-utans: central bias) in Experiment 2”. For clarification, add a sentence explaining what you mean. For example, something like “Specifically, rather than exploring the full range of possible locations, gorillas exhibited a strong preference for the left-most location and orangutans for the central locations.”

We made it more clear (lines 618-620).

Figure S3: there is no figure being displayed for me in the supplementary document, there is a white picture but nothing else
Fixed 

The authors have provided figures of individual performance in their response letter, but why not include them in the ESM for interested readers? For the figure of individual response Exp. 2: are error bars displayed correctly? It seems some values do have error bars while others don’t have them – or are they so small that they don’t display well? Its especially apparent in Figure C.
Indeed we fixed the error bars. The figures are now in the SM.

Reviewer #2: 

L 135. The authors stated in ref. 39 that bonobos chose the risky option in 87.5% of trials. The numbers do not seem to coincide with figure 2 of ref. 39 unless the authors are averaging between small and medium sizes. Is this the case? Please clarify.
We selected the trials in ref. 39 where the safe and the risky option had the same expected value (in this article, the indifference point is at EV=1). According to table 1 of ref. 39, this only corresponds to trials where the safe option was medium (V=3) and their was 3 risky cups (P=0.33). Going back to table 2 this corresponds to a level of risky choice of 75% (indeed, not 87,5%) for bonobos and 100% for chimpanzees.
 
L 274. Table 1 seems to miss a minus value "(-)" after EV 1.

Done

L 341: "we reached a model with interpretable terms".
Done

L 356: I believe the added predictor is a sixth rather than a third predictor in the model. Experiment 2 analysis contains the same 5 predictors of Experiment 1 (risk probability, risk value, specie, session and side of the safe cup) + the addition of type of reward.
We modified it (line 354).

L 360: There seems to be an interaction between the position of the cup and species, as indicated in the results and in Table 5. This information is missing here, together with the fact that species was also a predictor.
We modified it.

L 372: I am confused because the null models included the species variable. To my understanding, that means the authors did not analyze the effect of species statistically (a full model including species against a null model without the variable) in any of their models, and therefore species differences should not be reported.
Indeed species should not be kept in the null model. In any case, the comparison to the null model was removed (we tested the significance of predictors with analysis of deviance).

General comment: The results are more precise than in the previous version of the manuscript, but I still have considerations about how they are reported. The authors constantly report main effects even when interactions between those main effects are significant. This is especially salient in Experiment 1. Probability to win, value of the risky option and session are all significant. Similarly, the interactions between session * probability and session * value are also significant. The authors should only report the interaction' effects and plot the results accordingly. In other words, the predictors' effect only makes sense in interaction with each other, not on their own anymore.
Indeed, we modified the manuscript following your suggestion (see lines 395->405).

L 397: Please report more information on the significant two-way interaction. In which direction did they modify their choices?
See lines 395->405 and tables S4 and S5.

Table 3: The inclusion of tables is helpful, but I would suggest the authors report more information regarding degrees of freedom, confidence intervals (values that are already calculated since they are plotted), and the model's estimates.

See tables 3, 4, 5, 6 and tables  S3, S8.

L 431: "..we found that the the session"

Done

L 433: I would inform readers about the interaction between value and session. Also, I would suggest first reporting the effect of session in L 432 (that subjects increase their risk tendency over the experiment sessions) and then the non-significant effect of the interaction, clarifying that the apes did not learn the probabilities to win over repeated testing.
Modified following your suggestion (434->441).

L 465: Same general comment I made earlier. If an interaction between the position of the cup and species is significant, the position of the cup on the trolley per se adds no value to the interpretation of the results.
We modified it.

L 483: The significant interaction between the type of experiment and the value of the risky option is not reported in table 6. In addition, what do the authors exactly mean by "subjects were more attentive to the value of the risky option in exp 1 than 2". It is unclear that attentive means that apes chose the risky option less in exp 1 compared to exp 2, as shown in figure 4A
We were referring to the slope of the curve Choice = f(value risky option), that is steeper in Experiment 1 than in Experiment 2, suggesting that subjects were more attentive to variations of the value of risky option in Experiment 1. However, we removed that interpretation.

L 488; To clarify, trials with P = 1 refer to any trial with just two cups regardless of the number of rewards in the risky cup, right?
Yes, any trial with only the safe and one risky cup.

L 525: I would add that controlled for expected gains means when EV = 2.
Done

L 592: References missing for those experiments from experience providing partial feedback.
Done (line 581).

L 606: I would include the analysis in the manuscript. The authors reported the resulting plots to both reviewers in the previous review round. I think they should report the results of the analysis and the plots, at least in the supplementary materials, with a reference in the results section.
Done (lines 487 to 493 and figure S9).

L 686: I would say that there were no apparent differences between species since the authors previously report an almost significant effect of species (p = 0.052) in Experiment 1 (although the authors may not require reporting species differences if their effect was controlled for and thus not statistically analyzed).

Done (lines 673 to 675).
