# Supplementary material for: Rationality and cognitive bias in captive gorillas’ and orang-utans’ economic decision-making
Source: PLoS One. 2022 Dec 14;17(12):e0278150. doi: 10.1371/journal.pone.0278150 (PMC9749992; doi:10.1371/journal.pone.0278150)
Supplement: Attachment — Submitted filename: Review PLOS ONE 2022 round 3.docx [file pone.0278150.s007.docx]

The paper has improved and I am willing to accept the manuscript, after few minor comments and one clarification is addressed.

Main clarification point: I applaud the inclusion of the random slopes, the clarity in the interpretation of the results, and in general all statistical changes the authors have addressed. The methods and results sections are much clearer in the current version. However, although I do not think the following information is necessary to be included in an accepted version of the manuscript—I leave this decision to the editor, I would like to know why the authors did no longer use the full-null model comparison LRT method (as they do with full and final models) and instead tested the significance of their predictors with an analysis of deviance. Such a drastic change is worth an explanation before acceptance, especially when functions such as drop1 in lme4 package allows to obtain the p-values for single and interaction predictors with the LRT framework.

Besides, in L 376 there seems to be a typo: “with the response variable was the proportion”.
Also, in conclusions L 744 “are currently unknown are require..”
