# Supplementary material for: Rationality and cognitive bias in captive gorillas’ and orang-utans’ economic decision-making
Source: PLoS One. 2022 Dec 14;17(12):e0278150. doi: 10.1371/journal.pone.0278150 (PMC9749992; doi:10.1371/journal.pone.0278150)
Supplement: Attachment — Submitted filename: reviewers comment (round 3).odt [file pone.0278150.s008.odt]

Editor : 

- abstract: Pongo abelii should be in italics
- l 275: “Probability of obtaining the reward when choosing the risky option” rather than “Probability of winning the reward in the risky option”
ll 278-9: “each condition (box) consisted of 40 trials”
l 315: “each condition (box) consisted of 10 non-consecutive trials”
l 320: “we did not perform consecutively” (rather than “in a row”)
l 321: “was tested in two consecutive sessions”
Done

l 348: why was session number fitted as a categorical variable rather than a continuous variable?
Session was indeed fitted as a continuous variable, we corrected it.

l 376: “where the response variable was”
l 397: “showed that the main effects of the probability and the value of the risky options were significant (…) as well as those of the species (…) (please make a similar change also on ll 445-7 and 485-7)
ll 399 & 402: Actually, p = 0.051 or 0.052 are marginally significant, please rephrase on ll 399 and 402
l 413: please erase “that” before “the trend estimates”
l 447: “When investigating the effect of session on subjects’ choices”
l 456: “there was no difference” (please erase “specific”)
l 472: “left location” (please erase “most”)
l 486: please replace “the value to win” with “the value of the risky option”, here and wherever else applicable
l 497: “To investigate”
l 504: please erase “the” (typo)
ll 533-6: please add here a caveat concerning the small sample size of the present study
Done

l 539 Actually, capuchin monkeys were risk prone in this task (please check “F De Petrillo, M Ventricelli, G Ponsi, E Addessi 2015 Do tufted capuchin monkeys play the odds? Flexible risk preferences in Sapajus spp. Animal Cognition 18 (1), 119-130)
We specified it (line 107 and 531).


ll 577-8: please rephrase as follows: “decision-makers could experience probability distortions depending on how probabilities are presented to them”
l 581: “experiment based on experience”…”experiment based on description”
l 690: please erase “their” before “preliminary conclusions”

Done

Reviewer 1 :

I still can’t see a global model comparison to evaluate the contribution of the fixed predictor terms.  
We reverted to the LRT analysis in order to compare our full model to a null model (same random structure without fixed effects), then obtained a final model by removing non-significant interaction terms based on an analysis of deviance (type II Wald chisquare test).

The authors report they performed an analysis of deviance and did a model reduction by excluding random terms and nonsignificant fixed effects interaction terms. However, this process is not documented sufficiently. In l 340 it says „We removed non-significant interaction terms one by one until we reached a model with interpretable terms (without non-significant interaction terms)“. But this process is not sufficiently documented (which terms were removed; at what point/which order; based on comparison with which model/criterion?).

We made it more clear in the current version of the manuscript.

For example, in Table 3 we see that the three-way interactions are „significant“, but apparently some lower-level two-way interactions have been removed. It is unclear for me how the final model looked like.  When higher-level interactions are included in a model, lower-level interaction terms are automatically included as well. 
We made it more clear. For every analysis (and for the null, full and final model), the random and fixed structures are presented in the supplemental material.


The authors compare models with different random slopes structures, but I can’t see for what purpose this is done.  Generally, a maximum random slopes structure is recommended and for comparison of models to evaluate the fixed predictor terms, the compared models should have the same random slopes structure (Barr et al., 2013. https://doi.org/10.1016/j.jml.2012.11.001).
We did this in order to select the model with the more parsimonious random structure, but we removed that process in the current version and kept the complete random structure as you suggested.

Hence, what I would like to see is a comparison of the full model (fixed effects of interest [+ optional fixed effects of variables to only control for] + full random effect/slope structure) with a null model ([optional fixed effects to only control for] + full random effect/slope structure). If this comparison indicates that the full model doesn’t explain the data better than the null model, then that is the result. If this comparison indicates that the full model explains the data better than the null model, then the effects of the predictor terms can be assessed and potentially one continues to reduce fixed effects structure by removing nonsignificant interactions, starting with the highest-level interaction. But this global full-null model comparison is not reported, so we don’t even know if those significant terms even matter to explain the data better. See here, for example, for why model full model comparisons are important (Forstmeier & Schielzeth, 2011. 10.1007/s00265-010-1038-5 10.1007/s00265-010-1038-5).
We followed your suggestion and did this process for every analysis.

Why is effect of species tested as part of an interaction in Exp. 1 but not in Exp. 2?
The effect of species was tested as part of an interaction in both experiments but the effect was non-significant in Experiment 2 only, thus was removed from the final model. See supplemental tables S3 and S5 for the full models that we used in both experiments. 


L 355: „If needed we ran post-hoc tests to calculate estimated marginal means or estimated trends“. Could you specify what „if needed“ means, please.
We meant that if the main effect of a predictor (ex : session) was significant, we calculated EMM (for instance) for every level of that predictor. 


In general, I am a bit concerned that the authors might want too much of their data. Considering that only five resp. three individuals per species were tested, including two three-way-interactions and corresponding random slopes structure constitutes in a massively complex model! 
We added three-way interactions in order to answer questions from the reviewer in the previous rounds of reviews. The random slopes structure was also added at reviewers' suggestions. Would you wish that we simplify our models ?

I would be interested in information about effect sizes and model stability (i.e., comparing the estimates from the model based on all data with those from models with the levels of the random effects excluded one at a time). 
We followed your suggestion and selected the model with the more complete random structure.


L 388-391 „The final model without random slopes, and with individual and session within individual as random intercepts (see supplementary table S2) was not significantly different from the full model (LRT: χ2(4)=3.83, p=.43)“. This sounds circular to me. It’s intrinsic to the process of removing terms previously and seems kind of circular. When I have a model and remove stuff that is „not significant“ and then compare this reduced model to the previous model, it’s expected that the difference is not significant again (because this was the reason why the term was removed in the first place).
We followed your suggestion and selected the model with the more complete random structure.

L 340-341: Non-significant interaction terms should not be called „uninterpretable“.
Modified

Figures S6 and S7 are very busy. Would it be possible to split the data of the two species into two parallel plots respectively? In addition, it would help to have two different symbols representing the species level in addition to different line/symbol colours, wherever both species are presented in the same plot. And it would help if a colour legend would be displayed along with every Figure. The information given in Table S1 but it‘s very unintuitive having to look this up some pages down in a table.
Done

Fig. S4+S5: I would find it more intuitive to present session on the x-axis and display the value/probability to win of risky option as different lines.

Done

Reviewer #2

 I would like to know why the authors did no longer use the full-null model comparison LRT method (as they do with full and final models) and instead tested the significance of their predictors with an analysis of deviance. Such a drastic change is worth an explanation before acceptance, especially when functions such as drop1 in lme4 package allows to obtain the p-values for single and interaction predictors with the LRT framework.
We reverted to the LRT analysis in order to compare our full model to a null model (same random structure without fixed effects), then obtained a final model by removing non-significant interaction terms based on an analysis of deviance (type II Wald chisquare test).

Besides, in L 376 there seems to be a typo: “with the response variable was the proportion”.
Also, in conclusions L 744 “are currently unknown are require..”
Done
